# Coronary microvascular dysfunction is associated with poor glycemic control amongst female diabetics with chest pain and non-obstructive coronary artery disease

**DOI:** 10.1186/s12933-019-0833-1

**Published:** 2019-02-28

**Authors:** Jaskanwal D. Sara, Riad Taher, Nikhil Kolluri, Adrian Vella, Lilach O. Lerman, Amir Lerman

**Affiliations:** 1 Division of Cardiovascular Diseases and Department of Internal Medicine, Mayo College of Medicine, 200 First Street SW, Rochester, MN 55905 USA; 2 Internal Medicine Department and Department of Endocrinology, Rambam HealthCare Campus, Haifa, Israel; 3 Division of Internal Medicine, Mayo College of Medicine, Rochester, MN USA; 4 Division of Diabetes and Endocrinology, Mayo Clinic, Rochester, MN USA; 5 Division of Nephrology and Hypertension, Mayo Clinic, Rochester, MN USA

## Abstract

**Background:**

Patients with type 2 diabetes mellitus are at an increased risk of adverse cardiovascular events compared to those without diabetes. The timing, relative to disease onset, and degree of glycemic control that reduces the risk of adverse cardiovascular events remains uncertain. Coronary microvascular dysfunction is prevalent in patients with type 2 diabetes mellitus and is linked to adverse cardiovascular events. We assessed the association between endothelial-dependent and endothelial-independent coronary microvascular dysfunction and glycemic control in patients presenting with chest pain and nonobstructive coronary disease at angiography.

**Methods:**

Patients presenting with chest pain and found to have non-obstructive CAD (stenosis < 40%) at angiography underwent an invasive assessment of endothelial-independent and endothelial –dependent microvascular function. Endothelial-independent microvascular function was assessed by comparing the coronary flow velocity, measured using a Doppler guidewire, in response to intracoronary infusion of adenosine to calculate the coronary flow reserve ratio in response to adenosine (CFRAdn Ratio). A CFRAdn Ratio ≤ 2.5 was considered abnormal. Endothelial-dependent microvascular function was assessed by measuring the percent change in coronary blood flow in response to intracoronary infusions of acetylcholine (%ΔCBFAch), and microvascular endothelial dysfunction defined as a %ΔCBFAch of ≤ 50%. Patients were classified by normal versus abnormal CFRAdn Ratio and %ΔCBFAch. Measurements of HbA1c and fasting serum glucose were obtained prior to catheterization and compared between groups.

**Results:**

Between 1993 and 2012, 1469 patients (mean age 50.4 years, 35% male) underwent coronary angiography and invasive testing for coronary microvascular dysfunction, of which 129 (8.8%) had type 2 diabetes. Fifty-one (39.5%) had an abnormal %ΔCBFAch and 49 (38.0%) had an abnormal CFRAdn Ratio. Conventional cardiovascular risk factors and cardiovascular or diabetic medication use did not vary significantly between groups. Females with an abnormal CFRAdn Ratio or abnormal %ΔCBFAch had a significantly higher HbA1c compared to patients with a normal CFRAdn Ratio or %ΔCBFAch respectively: HbA1c % (standard deviation) 7.4 (2.1) vs. 6.5 (1.1), p = 0.035 and 7.3 (1.9) vs. 6.4 (1.2), p = 0.022, respectively. Female patients with an abnormal CFRAdn Ratio had significantly higher fasting serum glucose concentrations compared to those with a normal CFRAdn Ratio: fasting serum glucose mg/dL (standard deviation) 144.4 (55.6) vs. 121.9 (28.1), p = 0.035. This was not observed in men. Amongst female diabetics, a higher HbA1c was significantly associated with any coronary microvascular dysfunction both in a univariate and multivariate analysis: odds ratio (95% confidence interval) 1.69 (1.01–2.86) p = 0.049; and a fasting serum glucose > 140 mg/dL was significantly associated with an abnormal CFRAdn Ratio, 4.28 (1.43–12.81).

**Conclusion:**

Poor glycemic control is associated with coronary microvascular dysfunction amongst female diabetics presenting with chest pain and non-obstructive CAD. These findings highlight the importance of sex specific risk stratification models and treatment strategies when managing cardiovascular risk amongst diabetics. Further studies are required to identify additional risk prevention tools and therapies targeting microvascular dysfunction as an integrated index of cardiovascular risk.

**Electronic supplementary material:**

The online version of this article (10.1186/s12933-019-0833-1) contains supplementary material, which is available to authorized users.

## Introduction

Patients with type 2 diabetes mellitus are at an increased risk of adverse cardiovascular events compared to those without diabetes [1–3], and despite efforts at risk reduction including smoking cessation and blood pressure and cholesterol optimization, the majority of diabetics continue to die from cardiovascular disease [4]. Observational studies have shown a significant association between glycemic control and cardiovascular disease [5–8], however results from randomized clinical trials have been more controversial. The UK Prospective Diabetes Study (UKPDS) showed that HbA1c, was an independent predictor of cardiovascular outcomes [9]; the ADVANCE study showed that intensive glucose control (HbA1c < 6.5%) was associated with a reduction in major microvascular events, but not in major macrovascular events [10]; the VADT study showed that intensive glucose control had no significant effect on rates of major cardiovascular events, death or microvascular complications [11] while the ACCORD trial showed that aggressive glycemic control targeting normal HbA1c was associated with an increased mortality but did not reduce major cardiovascular events [12]. Thus, the role of glycemic control amongst diabetics in cardiovascular disease progression remains unclear.

Coronary microvascular dysfunction (CMD) is prevalent in patients with type 2 diabetes mellitus [13], and is characterized by pathologically attenuated microvascular vasorelaxation in response to increased demand. CMD is clinically meaningful as it mediates ischemia leading to angina [14, 15] and is associated with an increased risk of cardiovascular events [16, 17]. Studies have shown that systemic microvascular abnormalities may involve endothelin-1, which is common in patients with microvascular angina [18], while others have shown that impaired myocardial flow reserve is frequent in type 2 diabetics, and is strongly associated with the degree of albuminuria [19]. CMD and albuminuria may therefore share common mechanisms related to the pathogenesis of diabetic micro-vasculopathy. Microvascular complications are common in patients with type 2 diabetes and are related to disease duration and control [10], though the relationship between glycemic control and CMD is not well established.

Studies have shown that diabetes is a stronger risk factor for cardiovascular mortality in women compared to men [20, 21]. Differences in cardiovascular risk profile between men and women are well established. Women have fewer conventional cardiovascular risk factors compared to men and are more likely to experience cardiovascular events in the absence of obstructive CAD, which may be explained by a higher prevalence of functional vascular abnormalities such CMD and endothelial dysfunction [22–24]. We previously showed that hypothyroidism is associated with endothelial-dependent CMD amongst women and not men [25], and that elevated uric acid levels are associated with CMD and adverse outcomes in post-menopausal women [26]. Sex-based physiological differences may therefore play a role in vascular function and health, though differences in the relationship between coronary microvascular function and glycemic control across sexes in patients with diabetes is not well described. In the following study, we aim to compare the association between endothelial-dependent and endothelial-independent CMD and glycemic control between sexes in patients with diabetes who present with chest pain and non-obstructive CAD at coronary angiography. As a secondary aim, we aim to assess whether coronary microvascular function varies significantly depending on which cardiovascular and or diabetic medication(s) subjects are taking at the time of testing.

## Methods

### Study protocol

This retrospective cross-sectional study was approved by the Mayo Clinic Institutional Review Board. Patients were referred to our institution by their physician for assessment of chest pain. All patients were then evaluated by a cardiologist at our institution and those with signs or symptoms suspicious of stable cardiac ischemic as per the clinical assessment of the evaluating cardiologist and/or had an abnormal non-invasive stress test were then referred for a clinically indicated elective coronary angiogram. Patients with the following were excluded: greater than 40% diameter stenosis of any coronary artery; acute coronary syndrome; acute renal failure; uncontrolled hypertension; left ventricular ejection fraction of 50% or less and left ventricular hypertrophy.

Consecutive patients presented to the cardiac catheterization laboratory in the fasting state and all cardiovascular medications, including nitrates and calcium channel blockers, had been discontinued for at least 48 h. Routine diagnostic coronary angiography was performed on all patients using standard clinical protocols. Angiograms were reviewed prior to the infusion of any pharmacological agents. In cases where the severity of stenosis was uncertain, online quantitative coronary angiography was used. All patients underwent evaluation of microvascular endothelial-dependent and endothelial-independent coronary flow reserve as previously described [27, 28]. Following intravenous administration of 5000–7000 U of heparin, a Doppler guidewire (Flowire, Volcano) 0.014 inches in diameter within a 3-F. Slip-Cath Infusion Catheter (Cook Medical) was positioned into the mid-portion of the left anterior descending coronary artery, 2–3 mm distal to the tip of the infusion catheter. This vessel was chosen for accessibility and because it supplies the largest territory of the myocardium. Heart rate and mean arterial blood pressure were continuously monitored throughout each procedure [27, 29–31].

Baseline mean peak velocity was recorded using the intracoronary Doppler wire after which intracoronary bolus injections of increasing doses (18–72 µg) of adenosine, an endothelial-independent vasodilator affecting predominantly the microcirculation [32] was administered through the guide catheter until maximal hyperemia or a coronary flow reserve ratio > 2.5 had been achieved (see below) or the maximum dose of adenosine had been administered. The maximal mean peak velocity was then recorded and the endothelial-independent coronary flow velocity reserve ratio calculated by dividing the mean peak velocity following the administration of adenosine by the mean peak velocity at baseline (CFRAdn Ratio) [27, 29–31].

After a 5-min equilibration period, acetylcholine was infused at concentrations of 10^−6^, 10^−5^ and 10^−4^ M (to achieve estimated coronary bed concentrations of 10^−8^, 10^−7^ and 10^−6^ M respectively) for 3 min at each concentration to assess endothelial-dependent function as previously described [27–29]. Infusions were performed using a Harvard pump to maintain infusion rates of less than 1% of the estimated coronary blood flow (CBF). Doppler measurements of mean peak velocity were performed after each infusion followed by repeat coronary angiography. Coronary artery diameter was measured at baseline and after the infusion with acetylcholine, by an independent investigator blinded to Doppler velocity data using a previously described computer-based image analysis system [33, 34]. Endothelial-dependent CBF was then calculated using the following, as previously described [27, 29]: CBF = *π* (mean peak velocity/2)(coronary artery diameter/2)^2^. The maximal percentage increase in CBF in response to acetylcholine compared to the CBF at baseline was then calculated (%ΔCBFAch). For quality control, all measurements were performed in the segment 5 mm distal to the tip of the Doppler wire and following each infusion, the diameter was measured in the same segment of the vessel [27, 29–31].

### Definition of terms

CMD was defined as the presence of abnormal endothelial-independent coronary microvascular function and/or abnormal endothelial-dependent coronary microvascular function. Impaired endothelial-independent microvascular function was defined as a coronary flow velocity reserve ratio in response to adenosine (CFRAdn Ratio) of 2.5 or less [35]. Impaired endothelial-dependent microvascular function was defined as a maximal percentage increase in CBF in response to any dose of acetylcholine compared to baseline CBF (%ΔCBFAch) of 50% or less [30, 31, 36].

### Patient information

Data was collected on conventional cardiovascular risk factors including hypertension, diabetes mellitus, hyperlipidemia, smoking status and body mass index (BMI); biochemical parameters including fasting blood glucose, HbA1c, serum total cholesterol, low density lipoprotein (LDL), high density lipoprotein (HDL), and triglycerides; and medication use including antiplatelet and antihypertensive medication, statins as well as diabetic medication including use of insulin. Diabetes was defined as a documented history of diabetes, for which the diagnostic criteria required at least one of the following: a fasting serum glucose ≥ 126 mg/dL; serum glucose after a 75 g oral glucose tolerance test of ≥ 200 mg/dL after 2 h; a random serum glucose of ≥ 200 mg/dL in conjunction with symptoms of hyperglycemia that may include polydipsia, polyuria, polyphagia, fatigue, and weight loss; or an HbA1c ≥ 6.5% [37]. Smoking was categorized as a history of current smoking, former smoking or never smoking; hypertension was defined as a documented history of hypertension or receiving treatment with anti-hypertensives; and hyperlipidemia was defined as a history of total cholesterol levels of > 240 mg/dL or treatment with lipid-lowering therapy. All blood test results included in this study are based on blood samples obtained on the morning of the index procedure. A history of MI was also documented and was diagnosed in the presence of at least 2 of the following: (1) typical chest pain for at least 20 min; (2) raised creatinine kinase (or the MB fraction) or troponin level; (3) new ST-segment elevation, Q-waves or left bundle branch block on ECG. Information was also collected on past medical history including other vascular diseases (defined as a documented history of peripheral vascular disease, stroke or transient ischemic attack).

### Statistical analysis

Patients with type 2 diabetes mellitus were retrospectively identified and were categorized as having normal versus abnormal endothelial-independent microvascular function measured using the CFRAdn Ratio and normal versus abnormal endothelial-dependent microvascular function measured using %ΔCBFAch. Continuous variables are presented as a mean (standard deviation) where data is normally distributed and as a median (quartile 1, quartile 3) for skewed data. Categorical variables are presented as frequencies (percentages). Differences between groups were analyzed using Student’s T test and Wilcoxon rank-sum test for continuous variables and Pearson’s Chi squared test for proportions. Indices of glycemic control, namely HbA1c and fasting serum glucose concentrations, were compared as continuous variables between patients with normal versus abnormal endothelial-dependent and endothelial-independent microvascular function after stratifying all patients by sex. Univariate and multivariate logistic regression models were then fitted to assess the association between glycemic control and endothelial-dependent and endothelial-independent CMD. Indices of glycemic control included HbA1c and fasting serum glucose concentrations as continuous variables, and an HbA1c > 7% and a fasting serum glucose > 140 mg/dL as categorical variables. These thresholds were used to denote suboptimal glycemic control. Multivariate analyses were adjusted for conventional cardiovascular risk factors which could potentially confound the relationship between glycemic control and CMD and included age, BMI, total cholesterol, and systolic blood pressure at the time of cardiac catheterization as continuous variables, and smoking status as a categorical variable. A p-value less than 0.05 was considered significant and all statistical analyses were performed using JMP 9 software (SAS Institute, Inc., Cary, NC, USA).

## Results

### Sample overview

Between 1993 and 2012, 1469 patients (mean age 50.4 years, 35% male) underwent coronary angiography and invasive testing for CMD. One hundred twenty-nine patients had diabetes mellitus (8.8%) all of which were type 2. Patients were retrospectively divided into those with a normal versus abnormal CFRAdn Ratio and normal versus abnormal %ΔCBFAch. Forty-nine (38.0%) patients had an abnormal CFRAdn Ratio (CFR ratio of ≤ 2.5 in response to adenosine, characteristic of impaired endothelial-independent microvascular function). Fifty-one (39.5%) had an abnormal %ΔCBFAch (ΔCBF ≤ 50% in response to acetylcholine, characteristic of impaired endothelial-dependent microvascular function). Additionally, 93 subjects (72.1%) had any CMD (abnormal CFRAdn or abnormal %ΔCBFAch), and 34 (26.4%) had both an abnormal CFRAdn *and* abnormal %ΔCBFAch). Table 1 summarizes the baseline characteristics of all diabetic patients with normal versus abnormal CFRAdn Ratio and %ΔCBFAch. Patients with an abnormal %ΔCBFAch were significantly older than patients with a normal %ΔCBFAch: years (standard deviation) 56.2 (9.3) vs. 50.4 (10.9), p = 0.002. Age did not vary significantly between patients with a normal versus abnormal CFRAdn Ratio. More female patients had an abnormal CFRAdn Ratio compared to patients with a normal CFRAdn Ratio: 36 (73.5%) vs. 41 (51.3%), p = 0.011. The frequency of female patients did not vary significantly between patients with a normal versus abnormal %ΔCBFAch. There were no other significant differences between patients with normal versus abnormal CFRAdn Ratio or %ΔCBFAch with regards to the frequency of other cardiovascular risk factors, biochemical parameters, or vital signs. Patients with an abnormal CFRAdn Ratio had a significantly lower %ΔCBFAch compared to patients with a normal %ΔCBFAch: percentage (standard deviation) 20.7 (75.6) vs. 59.8 (97.8), p = 0.012. Patients with an abnormal %ΔCBFAch did not have a significant difference in CFRAdn Ratio compared to those with a normal %ΔCBFAch. Additional file 1: Table S1a summarizes the baseline characteristics of all diabetic patients stratified by sex with normal versus abnormal CFRAdn Ratio and %ΔCBFAch.

**Table 1:** Summary of clinical characteristics between patients with normal versus abnormal endothelial-independent and endothelial-dependent microvascular function

	CFRAdn Ratio > 2.5 N = 80 (62%)	CFRAdn Ratio ≤ 2.5 N = 49 (38%)	*p* value	%ΔCBFAch > 50% N = 78 (60.5%)	%ΔCBFAch ≤ 50% N = 51 (39.5%)	*p* value
Age, years (SD)	52.8 (10.1)	55.8 (10.4)	0.112	50.4 (10.9)	56.2 (9.3)	0.002
Female, n (%)	41 (51.3%)	36 (73.5%)	0.011	35 (68.6%)	42 (53.9%)	0.092
BMI, kg/m^2^ (SD)	33.4 (7.6)	33.9 (7.5)	0.726	33.0 (8.4)	34.0 (7.0)	0.497
Hypertension, n (%)	56 (70%)	30 (61.2)	0.307	32 (62.8)	54 (69.2)	0.446
Hyperlipidemia, n (%)	60 (75%)	37 (75.5%)	0.948	36 (70.6%)	61 (78.2%)	0.330
History of MI, n (%)	8 (10.0%)	8 (16.3%)	0.579	7 (13.7%)	9 (11.5%)	0.919
History of vascular disease, n (%)	10 (12.5%)	4 (8.2)	0.666	7 (13.7)	7 (9.0%)	0.597
Smoking status, n (%)						
Never smoked	31 (38.8%)	25 (51.0%)	0.439	17 (33.3%)	39 (50.0%)	0.152
Former smoker	39 (48.8%)	20 (40.8%)		29 (56.9%)	30 (38.5%)	
Current smoker	9 (11.3%)	4 (8.2%)		5 (9.8%)	8 (10.3%)	
Total cholesterol, mg/dL (SD)	179.5 (46.8)	178.7 (45.9)	0.922	183.2 (49.3)	176.6 (44.3)	0.452
HDL-C, mg/dL (SD)	48.6 (16.3)	47.5 (12.0)	0.679	49.3 (15.5)	47.4 (14.3)	0.499
LDL-C, mg/dL (SD)	98.0 (37.3)	95.8 (38.3)	0.762	100.5 (39.0)	95.0 (36.6)	0.444
Triglycerides, mg/dL (SD)	159.6 (103.7)	171.1 (116.2)	0.583	157.7 (114.5)	167.8 (104.3)	0.625
Insulin, mg/dL (SD)	16.4 (26.8)	36.0 (103.3)	0.265	16.1 (24.9)	28.7 (83.3)	0.278
BNP, pg/dL (SD)	57.3 (93.0)	66.8 (95.3)	0.695	55.7 (89.9)	64.4 (96.1)	0.717
Heart rate, bpm (SD)	70.5 (12.2)	74.7 (13.7)	0.085	72.6 (14.0)	71.8 (12.2)	0.748
Systolic blood pressure, mmHg (SD)	136.4 (19.1)	137.5 (21.1)	0.784	135.3 (21.3)	137.8 (19.0)	0.492
Diastolic blood pressure, mmHg (SD)	74.4 (12.8)	73.8 (15.4)	0.794	73.6 (14.2)	74.5 (13.7)	0.727
CFRAdn Ratio (SD)	3.2 (0.6)	2.2 (0.3)	< 0.001	2.9 (0.6)	2.8 (0.8)	0.682
%ΔCBFAch (SD)	59.8 (97.8)	20.7 (75.6)	0.012	130.7 (84.5)	− 11.1 (35.8)	< 0.001

BMI, body mass index; BNP, brain natriuretic peptide; CFRAdn Ratio, coronary flow reserve ratio in response to adenosine; HDL-C, high density lipoprotein cholesterol; LDL-C, low density lipoprotein cholesterol; MI, myocardial infarction; %ΔCBFAch, percentage change in coronary blood flow in response to acetylcholine

Table 2 summarizes the differences in the frequency of various medication use prior to coronary catheterization between patients with a normal versus abnormal CFRAdn Ratio and %ΔCBFAch. There were no significant differences in the frequency of use of cardiovascular medication or diabetic medication, including insulin, prior to catheterization between patients with a normal versus abnormal CFRAdn Ratio or between patients with a normal versus abnormal %ΔCBFAch. Additional file 1: Table S2a summarizes the differences in the frequency of medication use in patients after stratifying by sex.

**Table 2:** Summary of medication use at the time of coronary catheterization between patients with normal versus abnormal endothelial-independent and endothelial-dependent microvascular function

	CFRAdn Ratio > 2.5	CFRAdn Ratio ≤ 2.5	*p* value	%ΔCBFAch > 50%	%ΔCBFAch ≤ 50%	*p* value
Metformin, n (%)	28 (35.0%)	18 (36.7%)	0.842	16 (31.4%)	30 (38.5%)	0.409
Thiazolidinedione, n (%)	5 (6.3%)	4 (8.2%)	0.682	2 (3.9%)	7 (9.0%)	0.253
Sulfonylurea, n (%)	12 (15.0%)	9 (18.4%)	0.617	7 (13.7%)	14 (18.0%)	0.522
Meglitinides, n (%)	0 (0.0%)	1 (2.0%)	0.163	0 (0.0%)	1 (1.3%)	0.315
Dipeptidyl peptidase-4 inhibitors, n (%)	2 (2.5%)	0 (0%)	0.165	1 (2.0%)	1 (1.3%)	0.763
Glucagon-like peptide 1 analog, n (%)	3 (3.8%)	0 (0%)	0.088	2 (3.9%)	1 (1.3%)	0.337
Insulin, n (%)	16 (20%)	13 (26.5%)	0.392	11 (21.6%)	18 (23.1%)	0.841
Dihydropyridines, n (%)	15 (18.8%)	8 (16.3%)	0.726	9 (17.7%)	14 (18.0%)	0.965
Diltiazem, n (%)	18 (22.5%)	7 (14.3%)	0.244	14 (27.5%)	11 (14.1%)	0.063
Statins, n (%)	42 (52.5%)	25 (51.0%)	0.870	24 (47.1%)	43 (55.1%)	0.370
Ranolazine, n (%)	2 (2.5%)	1 (2.0%)	0.866	2 (3.9%)	1 (1.3%)	0.337
ACE-Inhibitors or ARBs, n (%)	36 (45.0%)	25 (51.0%)	0.506	21 (41.2%)	40 (51.3%)	0.260
Beta blockers, n (%)	31 (38.8%)	21 (42.9%)	0.645	20 (39.2%)	32 (41.0%)	0.838
Aspirin, n (%)	51 (63.8%)	34 (69.4%)	0.511	34 (66.7%)	51 (65.4%)	0.881
l-Arginine, n (%)	4 (5.0%)	2 (4.1%)	0.809	4 (7.8%)	2 (2.6%)	0.169
Diuretics, n (%)	22 (27.5%)	21 (42.9%)	0.074	14 (27.5%)	29 (37.2%)	0.249
Nitrates, n (%)	22 (27.5%)	16 (32.7%)	0.535	17 (33.3%)	21 (26.9%)	0.437

ACE-inhibitors, angiotensin converting enzyme-inhibitors; ARB, angiotensin receptor blockers; CFRAdn Ratio, coronary flow reserve ratio in response to adenosine; %ΔCBFAch, percentage change in coronary blood flow in response to acetylcholine

### Diabetic control and microvascular function—univariate analyses

**Fig. 1:**
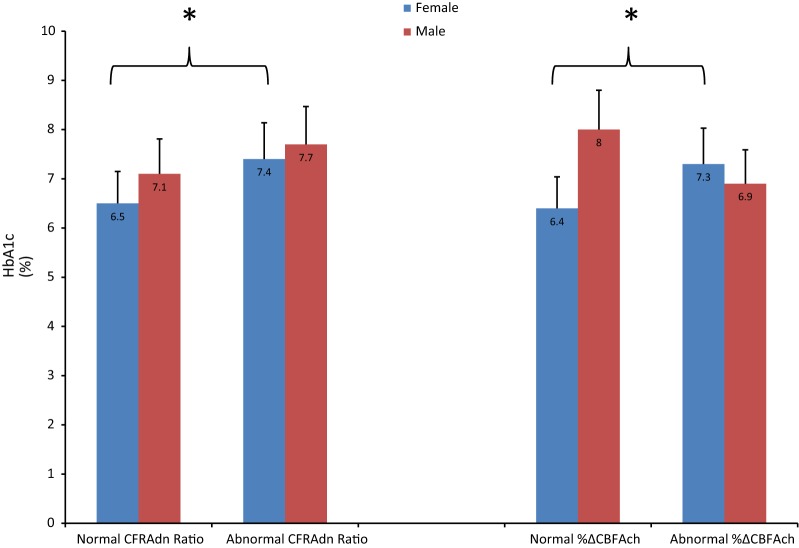
Bar graph outlining the differences in HbA1c levels between patients with a normal versus abnormal coronary flow reserve ratio in response to adensoine, and normal versus abnormal percentage change in coronary blood flow in response to acetylcholine after startifying by sex. CFRAdn Ratio, coronary flow reserve ratio in response to adenosine; %ΔCBFAch, percentage change in coronary blood flow in response to acetylcholine. *p < 0.05, bar lines represent standard deviations. An HbA1c > 7% correlates with suboptimal glycemic control

**Fig. 2:**
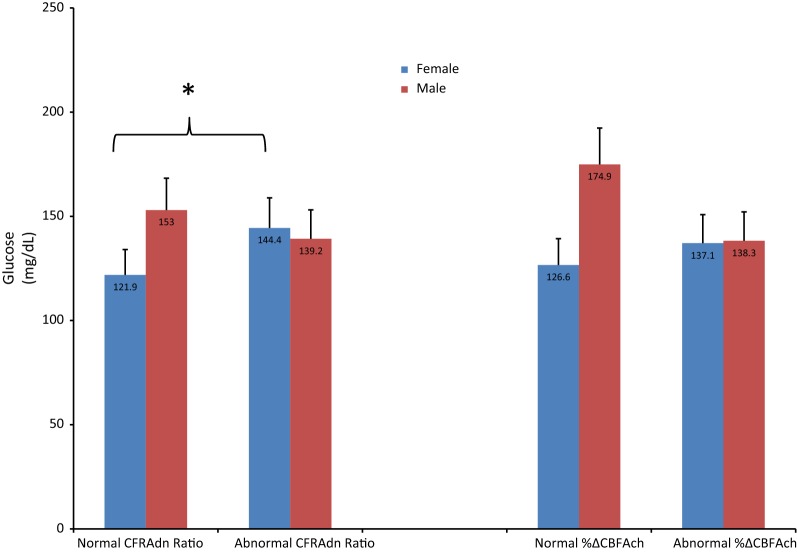
Bar graph outlining the differences in fasting serum gluocose levels (mg/dL) between patients with a normal versus abnormal coronary flow reserve ratio in response to adenosine, and normal versus abnormal percentage change in coronary blood flow in response to acetylcholine after startifying by sex. CFRAdn Ratio, coronary flow reserve ratio in response to adenosine; %ΔCBFAch, percentage change in coronary blood flow in response to acetylcholine. *p < 0.05, bar lines represent standard deviations. A fasting serum glucose > 140 mg/dL correlates with suboptimal glycemic control

Figure 1 shows the differences in HbA1c at the time of coronary catheterization between patients with a normal versus abnormal CFRAdn Ratio, and a normal versus abnormal %ΔCBFAch after stratifying all patients by sex. Females with an abnormal CFRAdn Ratio had a significantly higher HbA1c compared to patients with a normal CFRAdn Ratio, as did females with an abnormal %ΔCBFAch compared to those with a normal %ΔCBFAch: HbA1c (standard deviation) 7.4% (2.1) vs. 6.5% (1.1), p = 0.035 and 7.3% (1.9) vs. 6.4% (1.2), p = 0.022, respectively. Amongst males, HbA1c did not vary significantly between groups. Figure 2 shows the differences in fasting serum glucose (mg/dL) concentrations at the time of coronary catheterization between patients with a normal versus abnormal CFRAdn Ratio, and normal versus abnormal %ΔCBFAch after stratifying all patients by sex. Female patients with an abnormal CFRAdn Ratio had a significantly higher fasting serum glucose level compared to those with a normal CFRAdn Ratio: fasting serum glucose (standard deviation) 144.4 mg/dL (55.6) vs. 121.9 mg/dL (28.1), p = 0.035. Fasting serum glucose levels did not vary significantly between female patients with an abnormal versus normal %ΔCBFAch. Fasting serum glucose levels did not vary significantly between male patients with a normal versus abnormal CFRAdn Ratio or normal versus abnormal %ΔCBFAch.

Table 3 summarizes the association between diabetic control and endothelial-independent and endothelial-dependent CMD in patients stratified by sex. Diabetic control was characterized as a continuous variable with HbA1c (%) and fasting serum glucose (mg/dL) at the time of coronary catheterization, and was also categorized into binary variables denoting suboptimal glycemic control with an HbA1c > 7% and a fasting serum glucose > 140 mg/dL. Amongst female patients with type 2 diabetes, HbA1c as a continuous variable was significantly associated with an abnormal CFRAdn Ratio and an abnormal %ΔCBFAch: odds ratio (confidence interval) 1.47 (1.02–2.13) p = 0.040 and 1.52 (1.02–2.27) p = 0.038, respectively. However, after stratifying female patients on the basis of their HbA1c with an HbA1c > 7% correlating with suboptimal glycemic control, an HbA1c > 7% was not significantly associated with either an abnormal CFRAdn Ratio or an abnormal %ΔCBFAch. Fasting serum glucose was significantly associated with an abnormal CFRAdn Ratio amongst female patients: odds ratio (confidence interval) 1.02 (1.00–1.03) p = 0.035, though was not significantly associated with an abnormal %ΔCBFAch. After stratifying female patients on the basis of their fasting serum glucose levels with a fasting glucose > 140 mg/dL correlating with suboptimal glycemic control, a fasting glucose > 140 mg/dL was significantly associated with an abnormal CFRAdn Ratio: odds ratio (confidence interval) 3.36 (1.24–9.07) p = 0.017, though was not significantly associated with an abnormal %ΔCBFAch.

**Table 3:** Univariate analyses of the relationship between glycemic control and abnormal endothelial-independent and endothelial-dependent microvascular function

Odds ratio (95% confidence interval)	Abnormal CFRAdn Ratio	p value	Abnormal %ΔCBFAch	p value
*Females*				
HbA1c (per unit change)	1.47 (1.02–2.13)	0.040	1.52 (1.02–2.27)	0.038
HbA1c > 7%	1.60 (0.59–4.38)	0.357	1.60 (0.58–4.41)	0.364
Glucose (per unit change)	1.02 (1.00–1.03)	0.035	1.01 (0.99–1.02)	0.312
Glucose > 140 mg/dL	3.36 (1.24–9.07)	0.017	1.60 (0.62–4.30)	0.340
*Males*				
HbA1c (per unit change)	1.20 (0.84–1.72)	0.315	0.67 (0.46–1.00)	0.049
HbA1c > 7%	1.77 (0.47–6.62)	0.397	0.29 (0.08–1.03)	0.056
Glucose (per unit change)	1.00 (0.98–1.01)	0.527	0.99 (1.00–1.01)	0.111
Glucose > 140 mg/dL	1.37 (0.39–4.89)	0.625	0.39 (0.11–1.29)	0.125

CFRAdn Ratio, coronary flow reserve ratio in response to adenosine; %ΔCBFAch, percentage change in coronary blood flow in response to acetylcholine

Amongst males, HbA1c was negatively associated with an abnormal %ΔCBFAch with borderline significance: odds ratio (confidence interval) 0.67 (0.46–1.00) p = 0.049, though was not significantly associated with an abnormal CFRAdn Ratio. An HbA1c > 7%, and fasting serum glucose, as a continuous variable or as a categorical variable (fasting serum glucose > 140 mg/dL) were not significantly associated with an abnormal CFRAdn Ratio or %ΔCBFAch in diabetic men.

### Diabetic control and microvascular function—multivariate analyses

Table 4 summarizes the association between diabetic control and endothelial-independent and endothelial-dependent CMD in patients stratified by sex, after adjusting for age, body mass index, smoking status, total cholesterol and systolic blood pressure at the time of catheterization. Amongst female diabetics, HbA1c as a continuous variable was significantly associated with an abnormal CFRAdn Ratio, odds ratio (95% confidence interval) 1.61 (1.07–2.40) p = 0.021; abnormal %ΔCBFAch, 1.51 (1.00–2.28) p = 0.048; and any CMD, 1.69 (1.01–2.86) p = 0.049. However, an HbA1c > 7 was not significantly associated with any type of CMD. Fasting serum glucose as a continuous variable was significantly associated with an abnormal CFRAdn Ratio, 1.02 (1.01–1.04) p = 0.014 but not with an abnormal %ΔCBFAch. A fasting serum glucose > 140 mg/dL was significantly associated with an abnormal CFRAdn Ratio, 4.28 (1.43–12.81) but not with an abnormal %ΔCBFAch. Amongst male patients with type 2 diabetes, HbA1c was negatively associated with an abnormal %ΔCBFAch, odds ratio (confidence interval) 0.29 (0.09–0.92) p = 0.036 and, but was not associated with any other dependent variable. Fasting serum glucose was not significantly associated with any dependent variable amongst diabetic males.

**Table 4:** Multivariate analyses of the relationship between glycemic control and abnormal endothelial-independent and endothelial-dependent microvascular function

Odds ratio (95% confidence interval)	Abnormal CFRAdn Ratio	p value	Abnormal %ΔCBFAch	p value
*Females*				
HbA1c (per unit change)	1.61 (1.07–2.40)	0.021	1.51 (1.00–2.28)	0.048
HbA1c > 7%	2.13 (0.71–6.38)	0.175	1.65 (0.55–4.95)	0.375
Glucose (per unit change)	1.02 (1.00–1.04)	0.014	1.00 (0.99–1.02)	0.846
Glucose > 140 mg/dL	4.28 (1.43–12.81)	0.009	1.54 (0.52–4.55)	0.434
*Males*				
HbA1c (per unit change)	1.51 (0.90–2.54)	0.121	0.29 (0.09–0.92)	0.036
HbA1c > 7%	3.96 (0.62–25.14)	0.145	0.11 (0.02–0.75)	0.025
Glucose (per unit change)	1.01 (0.99–1.02)	0.423	0.99 (0.97–1.01)	0.289
Glucose > 140 mg/dL	4.16 (0.71–24.22)	0.113	0.23 (0.04–1.34)	0.101

Multivariate anaylsis adjusted for the following variables: age, body mass index, smoking status, total cholesterol and systolic blood pressure at the time of catheterization

CFRAdn Ratio, coronary flow reserve ratio in response to adenosine; %ΔCBFAch, percentage change in coronary blood flow in response to acetylcholine

## Discussion

### Summary of findings

In the current study we show that CMD is prevalent amongst an unselected population of type 2 diabetics who present with chest pain and non-obstructive CAD at coronary angiography. Patients with endothelial-dependent CMD were significantly older than those with normal microvascular endothelial function, and patients with endothelial-independent CMD were more likely to be female than those with normal endothelial-independent microvascular function. We also showed that HbA1c was significantly higher in females with diabetes who had endothelial-independent and endothelial-dependent CMD, an association not seen in men, and that fasting serum glucose levels were significantly higher amongst females with endothelial-independent CMD. Lastly, amongst females with diabetes HbA1c was significantly associated with endothelial-independent and endothelial-dependent CMD separately, and a fasting serum glucose ≥ 140 mg/dL was significantly associated with endothelial-independent CMD even after adjusting for confounders. These findings suggest a link between glycemic control and functional coronary microvascular abnormalities in females with diabetes, and may implicate CMD as a potential mediator of ischemia in diabetics with suboptimal glycemic control.

### Glycemic control and the risk of micro- and macrovascular complications

Patients with type 2 diabetes mellitus are at an increased risk of angina and adverse cardiovascular events compared to those without diabetes [1–3], and despite efforts at risk reduction the majority of diabetics continue to die from cardiovascular disease [4]. Previous studies have shown that CMD is common in patients with type 2 diabetes [13], and in the current study we show that 72.1% of diabetics had some sort of CMD. Both endothelial-dependent and -independent CMD are linked to ischemia [14, 15], and thus CMD could represent the underlying mechanism for angina in diabetics who have non-obstructive CAD at angiography. Studies have also shown that systemic microvascular abnormalities may involve endothelin-1 and are common in patients with microvascular angina [18], while others have shown that impaired myocardial flow reserve, which leads to angina, is frequent in type 2 diabetics, and is strongly associated with the degree of albuminuria [19]. These findings suggest that CMD and albuminuria might share common mechanisms, and underscores the notion that microvascular disease in diabetes is a systemic phenomenon extending beyond ‘known’ microvascular beds such as the kidneys and into the coronary circulation.

Further, observational studies [5–8] have shown a significant association between glycemic control and cardiovascular disease. Results from randomized clinical trials however have been more controversial. The UK Prospective Diabetes Study (UKPDS) showed that hyperglycemia assessed by HbA1c was an predictor of cardiovascular disease [9]; the ADVANCE study showed that intensive glucose control (HbA1c < 6.5%) was associated with a reduction in major microvascular events, driven primarily by reduction in nephropathy, but not major macrovascular events [10]; the VADT study showed that intensive glucose control had no significant effect on rates of major cardiovascular events, death or microvascular complications [11], while the ACCORD trial showed that aggressive glycemic control targeting normal HbA1c was associated with an increased mortality and did not reduce major cardiovascular events [12]. In the current study we showed that after adjusting for covariates HbA1c was associated with any CMD, and a fasting serum glucose > 140 mg/dL was associated with endothelial-independent CMD in females but not males with type 2 diabetes. The differences observed in the aforementioned clinical trials may, in part, be explained by differences in the number of males and females in each study, particularly as the ADVANCE, VADT and ACCORD trials all included a higher proportion of male subjects and did not show a significant association between ‘optimal glycemic control’ and improved cardiovascular risk. As CMD is common in diabetes and has been linked to adverse cardiovascular events, particularly among females [16, 17], the potential link between glycemic control and cardiovascular morbidity and mortality could be explained and mediated, in part, by CMD. This however requires further investigation with prospective studies. Thus, risk prevention strategies in type 2 diabetics could include therapies specifically targeted at CMD.

### Therapies to improve CMD

In the current study we showed that cardiovascular medication use, including statins, and vasoactive drugs such as beta blockers, calcium channel blockers, ACE-inhibitors and long-acting nitrates were not significantly associated with endothelial-dependent or -independent CMD. Whether these medications alter coronary microvascular function per se has not been consistently shown, though studies have indicated potential symptomatic value of various cardiovascular drugs in patients with CMD [38]. Thus given these potential benefits, therapeutic trials of beta-blockers, calcium channel blockers, ACE-inhibitors and statins have all been recommended in cases in which there are no contraindications. In addition, we showed that use of antidiabetic drugs, including insulin, was not associated with CMD. A previous study showed that women taking metformin not only experienced reductions in weight and improvements in insulin resistance but also had improvement in endothelial-dependent microvascular function and incidence of chest pain [39]. Other studies showed a potential association between exogenously administered insulin and impaired endothelial dysfunction [40]. The current study however does not support a relationship between diabetic medication and the prevalence of CMD in patients with type 2 diabetes, though we were limited by small sample sizes. Further studies are required to better clarify the relationship between CMD and pharmacologic therapy and the best approach to managing these patients.

### Sex-specific differences in cardiovascular risk

Differences in cardiovascular risk profile between men and women are well established. Women have fewer conventional cardiovascular risk factors compared to men and are more likely to experience cardiovascular events in the absence of obstructive CAD, which may be explained by a higher prevalence of functional vascular abnormalities such endothelial dysfunction and microvascular disease [22–24]. Further, studies have shown that diabetes is a stronger risk factor for cardiovascular mortality in women compared to men [20, 41]. The current study suggests that glycemic control is associated with CMD in female but not male diabetics highlighting a further potential difference in clinical profiles between sexes. We previously showed that hypothyroidism is associated with endothelial-dependent CMD amongst women and not men [25] and that elevated uric acid levels are associated with CMD and adverse outcomes amongst post-menopausal women [26]. Sex-based physiological differences likely modify the pathologic effects of a variety of metabolic stressors, including potentially the role of glycemic control in diabetics. Thus the current study adds to the growing body of evidence supporting the need for sex-specific risk management strategies in cardiovascular medicine that address the clinically distinct risk profile that women have compared to men. Further, female sexual hormones and menstrual cycle differences can contribute to vascular functional differences and could therefore influence the prevalence and severity of CMD at the time of invasive pharmacologic provocation testing. In the current study we did not ascertain the stage of menstrual cycle in the female subjects included in this study, though this could represent a potentially interesting additional step for future studies.

### Measures of good diabetic control

As to which index of ‘optimal glycemic control’ is most important has remained an area of controversy. The current study shows that linear increments of HbA1c are associated with any CMD. Conversely, fasting serum glucose as a continuous variable was not associated with any dependent variable, but a fasting serum glucose > 140 mg/dL was associated with endothelial-independent CMD. HbA1c is an accepted reliable marker of overall, longer term glycemic control integrating fasting and postprandial states [42] as well as mean glucose levels [43], and may therefore be more strongly related to different facets of vascular health such as endothelial cell function than fasting serum glucose levels alone, which form a less comprehensive index of glycemic. Nevertheless the current clinically accepted target of an HbA1c of 7% or less did not correlate with CMD in the current study, which is in keeping with other studies that have suggested that better targets for optimal glycemic control should be identified [42, 44, 45]. For example, it has been shown that long-term visit-to-visit glycemic variability is an additional and frequently a better glycemic parameter than mean HbA1c concentrations for assessing the risk of future development of micro- and macrovascular complications in patients with type 2 diabetes [46], though it may limited by its cumbersome methodology. Indeed a good approach could include techniques that preferentially assess vascular health, which could provide an integrated index of the cumulative effects of vasculo-protective factors as well as harmful factors, including sub-optimal glycemic control, and in doing so could offer clinical value above and beyond a single blood test. Further clinical studies are required to evaluate the potential utility of these tools.

### Study limitations

This study has a number of limitations. First, the study population consists of type 2 diabetics presenting with chest pain who were referred for coronary angiography to a tertiary referral center, and so the prevalence, severity and reversibility of CMD may be different to other populations. Further, even though all patients in this study were referred for a clinically indicated coronary angiogram having presented with signs and symptoms suspicious for stable cardiac ischemia based on the clinical evaluation of a cardiologist at our institution, some patients in this group may ultimately have had non-cardiac chest pain as opposed to stable angina. Second, the cross-sectional design of this study makes determining a causal association between glycemic control and CMD not possible and also prevented us from evaluating the impact of temporal changes in glycemic control as well as the incidence of cardiovascular events in patients with type 2 diabetes. Equally we cannot show that CMD is a mechanism for the incidence of cardiovascular events in patients with diabetes, as hypothesized in this study. Thus the current study is hypothesis-generating and this question would be better investigated in prospective clinical trials. Third, some of our findings may be limited by relatively small sample sizes. Fourth, HbA1c and fasting glucose levels were only measured once prior to cardiac catheterization and so may not give a complete picture of glycemic control or the severity of glucose variability that has a strong role in cardiovascular risk in diabetic patients. Similarly, we did not collect data on the duration of diabetes, the presence of diabetic-related complications, or on renal parameters such as urine albumin creatinine ratios, all of which can influence cardiovascular risk and could therefore confound the potential association between glycemic control and coronary microvascular function.

## Conclusion

Poor glycemic control is associated with CMD in female diabetics presenting with chest pain and non-obstructive CAD. These findings highlight the importance of sex-specific risk stratification models and treatment strategies when managing cardiovascular risk amongst diabetics. Further studies are required to identify additional risk prevention tools and therapies targeting CMD as an integrated index of cardiovascular risk.

## Additional file

**Additional file 1: Table S1.** Summary of clinical characteristics between patients stratified by sex with normal versus abnormal endothelial-independent and endothelial-dependent microvascular function. **Table S2.** Summary of medication use at the time of coronary catheterization between patients stratified by sex with normal versus abnormal endothelial-independent and endothelial-dependent microvascular function.

## Abbreviations

BMI: body mass index
CAD: coronary artery disease
CBF: coronary blood flow
CFR: coronary flow reserve
CMD: coronary microvascular dysfunction
HDL: high density lipoprotein
LDL: low density lipoprotein
MI: myocardial infarction

## References

[1] Huxley R, Barzi F, Woodward M (2006). Excess risk of fatal coronary heart disease associated with diabetes in men and women: meta-analysis of 37 prospective cohort studies. BMJ.

[2] Sarwar N, Gao P, Seshasai SR, Gobin R, Kaptoge S, Emerging Risk Factors C (2010). Diabetes mellitus, fasting blood glucose concentration, and risk of vascular disease: a collaborative meta-analysis of 102 prospective studies. Lancet.

[3] Roger VL, Go AS, Lloyd-Jones DM, Adams RJ, Berry JD, Brown TM (2011). Heart disease and stroke statistics—2011 update: a report from the American Heart Association. Circulation.

[4] Gu K, Cowie CC, Harris MI (1998). Mortality in adults with and without diabetes in a national cohort of the US population, 1971–1993. Diabetes Care.

[5] Khaw KT, Wareham N, Bingham S, Luben R, Welch A, Day N (2004). Association of hemoglobin A1c with cardiovascular disease and mortality in adults: the European prospective investigation into cancer in Norfolk. Ann Intern Med.

[6] Selvin E, Marinopoulos S, Berkenblit G, Rami T, Brancati FL, Powe NR (2004). Meta-analysis: glycosylated hemoglobin and cardiovascular disease in diabetes mellitus. Ann Intern Med.

[7] Lawes CM, Parag V, Bennett DA, Suh I, Lam TH, Whitlock G (2004). Blood glucose and risk of cardiovascular disease in the Asia Pacific region. Diabetes Care.

[8] Tancredi M, Rosengren A, Svensson AM, Kosiborod M, Pivodic A, Gudbjornsdottir S (2015). excess mortality among persons with type 2 diabetes. N Engl J Med.

[9] Chalmers J, Cooper ME (2008). UKPDS and the legacy effect. N Engl J Med.

[10] Patel A, MacMahon S, Chalmers J, Neal B, Billot L, Group AC (2008). Intensive blood glucose control and vascular outcomes in patients with type 2 diabetes. N Engl J Med.

[11] Duckworth W, Abraira C, Moritz T, Reda D, Emanuele N, Reaven PD (2009). Glucose control and vascular complications in veterans with type 2 diabetes. N Engl J Med.

[12] Gerstein HC, Miller ME, Byington RP, Goff DC, Bigger JT, Action to Control Cardiovascular Risk in Diabetes Study G (2008). Effects of intensive glucose lowering in type 2 diabetes. N Engl J Med.

[13] Picchi A, Capobianco S, Qiu T, Focardi M, Zou X, Cao JM (2010). Coronary microvascular dysfunction in diabetes mellitus: a review. World J Cardiol..

[14] Cheng TO, Bashour T, Kelser GA, Weiss L, Bacos J (1973). Variant angina of Prinzmetal with normal coronary arteriograms. A variant of the variant. Circulation..

[15] Phan A, Shufelt C, Merz CN (2009). Persistent chest pain and no obstructive coronary artery disease. JAMA.

[16] Pepine CJ, Anderson RD, Sharaf BL, Reis SE, Smith KM, Handberg EM (2010). Coronary microvascular reactivity to adenosine predicts adverse outcome in women evaluated for suspected ischemia results from the National Heart, Lung and Blood Institute WISE (Women’s Ischemia Syndrome Evaluation) study. J Am Coll Cardiol.

[17] Halcox JP, Schenke WH, Zalos G, Mincemoyer R, Prasad A, Waclawiw MA (2002). Prognostic value of coronary vascular endothelial dysfunction. Circulation.

[18] Ford TJ, Rocchiccioli P, Good R, McEntegart M, Eteiba H, Watkins S (2018). Systemic microvascular dysfunction in microvascular and vasospastic angina. Eur Heart J.

[19] Potier L, Chequer R, Roussel R, Mohammedi K, Sismail S, Hartemann A (2018). Relationship between cardiac microvascular dysfunction measured with 82Rubidium-PET and albuminuria in patients with diabetes mellitus. Cardiovasc Diabetol..

[20] Lee WL, Cheung AM, Cape D, Zinman B (2000). Impact of diabetes on coronary artery disease in women and men: a meta-analysis of prospective studies. Diabetes Care.

[21] Nedkoff L, Knuiman M, Hung J, Briffa TG (2016). Long-term all-cause and cardiovascular mortality following incident myocardial infarction in men and women with and without diabetes: temporal trends from 1998 to 2009. Eur J Prev Cardiol..

[22] Redberg RF, Cannon RO, Bairey Merz N, Lerman A, Reis SE, Sheps DS (2004). Women’s ischemic syndrome evaluation: current status and future research directions: report of the National Heart, Lung and Blood Institute workshop: October 2–4, 2002: Section 2: stable ischemia: pathophysiology and gender differences. Circulation.

[23] Zeiher AM, Drexler H, Wollschlager H, Just H (1991). Endothelial dysfunction of the coronary microvasculature is associated with coronary blood flow regulation in patients with early atherosclerosis. Circulation.

[24] Sara JD, Widmer RJ, Matsuzawa Y, Lennon RJ, Lerman LO, Lerman A (2015). Prevalence of coronary microvascular dysfunction among patients with chest pain and nonobstructive coronary artery disease. JACC Cardiovasc Interv..

[25] Sara JD, Zhang M, Gharib H, Lerman LO, Lerman A (2015). Hypothyroidism is associated with coronary endothelial dysfunction in women. J Am Heart Assoc..

[26] Prasad M, Matteson EL, Herrmann J, Gulati R, Rihal CS, Lerman LO (2017). Uric acid is associated with inflammation, coronary microvascular dysfunction, and adverse outcomes in postmenopausal women. Hypertension.

[27] Hasdai D, Cannan CR, Mathew V, Holmes DR, Lerman A (1996). Evaluation of patients with minimally obstructive coronary artery disease and angina. Int J Cardiol.

[28] Lerman A, Holmes DR, Bell MR, Garratt KN, Nishimura RA, Burnett JC (1995). Endothelin in coronary endothelial dysfunction and early atherosclerosis in humans. Circulation.

[29] Hasdai D, Gibbons RJ, Holmes DR, Higano ST, Lerman A (1997). Coronary endothelial dysfunction in humans is associated with myocardial perfusion defects. Circulation.

[30] Hasdai D, Holmes DR, Higano ST, Burnett JC, Lerman A (1998). Prevalence of coronary blood flow reserve abnormalities among patients with nonobstructive coronary artery disease and chest pain. Mayo Clin Proc.

[31] Widmer RJ, Flammer AJ, Herrmann J, Rodriguez-Porcel M, Wan J, Cohen P (2013). Circulating humanin levels are associated with preserved coronary endothelial function. Am J Physiol Heart Circulatory Physiol.

[32] Hori M, Kitakaze M (1991). Adenosine, the heart, and coronary circulation. Hypertension.

[33] Bell MR, Britson PJ, Chu A, Holmes DR, Bresnahan JF, Schwartz RS (1997). Validation of a new UNIX-based quantitative coronary angiographic system for the measurement of coronary artery lesions. Cathet Cardiovasc Diagn.

[34] Bove AA, Holmes DR, Owen RM, Bresnahan JF, Reeder GS, Smith HC (1985). Estimation of the effects of angioplasty on coronary stenosis using quantitative video angiography. Cathet Cardiovasc Diagn.

[35] Serruys PW, di Mario C, Piek J, Schroeder E, Vrints C, Probst P (1997). Prognostic value of intracoronary flow velocity and diameter stenosis in assessing the short—and long-term outcomes of coronary balloon angioplasty: the DEBATE Study (Doppler Endpoints Balloon Angioplasty Trial Europe). Circulation.

[36] Wei J, Mehta PK, Johnson BD, Samuels B, Kar S, Anderson RD (2012). Safety of coronary reactivity testing in women with no obstructive coronary artery disease: results from the NHLBI-sponsored WISE (Women’s Ischemia Syndrome Evaluation) study. JACC Cardiovasc Interven.

[37] American Diabetes A (2018). Classification and diagnosis of diabetes: standards of medical care in diabetes-2018. Diabetes Care.

[38] Ong P, Athanasiadis A, Sechtem U (2015). Pharmacotherapy for coronary microvascular dysfunction. Eur Heart J Cardiovasc Pharmacother..

[39] Jadhav S, Ferrell W, Greer IA, Petrie JR, Cobbe SM, Sattar N (2006). Effects of metformin on microvascular function and exercise tolerance in women with angina and normal coronary arteries: a randomized, double-blind, placebo-controlled study. J Am Coll Cardiol.

[40] Arcaro G, Cretti A, Balzano S, Lechi A, Muggeo M, Bonora E (2002). Insulin causes endothelial dysfunction in humans: sites and mechanisms. Circulation.

[41] Orchard TJ (1996). The impact of gender and general risk factors on the occurrence of atherosclerotic vascular disease in non-insulin-dependent diabetes mellitus. Ann Med.

[42] Colette C, Monnier L (2007). Acute glucose fluctuations and chronic sustained hyperglycemia as risk factors for cardiovascular diseases in patients with type 2 diabetes. Horm Metab Res.

[43] Rohlfing CL, Wiedmeyer HM, Little RR, England JD, Tennill A, Goldstein DE (2002). Defining the relationship between plasma glucose and HbA(1c): analysis of glucose profiles and HbA(1c) in the diabetes control and complications trial. Diabetes Care.

[44] Brownlee M, Hirsch IB (2006). Glycemic variability: a hemoglobin A1c-independent risk factor for diabetic complications. JAMA.

[45] Giorgino F, Leonardini A, Laviola L (2013). Cardiovascular disease and glycemic control in type 2 diabetes: now that the dust is settling from large clinical trials. Ann N Y Acad Sci.

[46] Cardoso CRL, Leite NC, Moram CBM, Salles GF (2018). Long-term visit-to-visit glycemic variability as predictor of micro- and macrovascular complications in patients with type 2 diabetes: the Rio de Janeiro type 2 diabetes cohort study. Cardiovasc Diabetol..

