# Supplementary material for: Coronary microvascular dysfunction is associated with poor glycemic control amongst female diabetics with chest pain and non-obstructive coronary artery disease
Source: Cardiovasc Diabetol. 2019 Feb 28;18:22. doi: 10.1186/s12933-019-0833-1 (PMC6393964; doi:10.1186/s12933-019-0833-1)
Supplement: Supplementary file 1 — Additional file 1: Table S1. Summary of clinical characteristics between patients stratified by sex with normal versus abnormal endothelial-independent and endothelial-dependent microvascular function. Table S2. Summary of medication use at the time of coronary catheterization between patients stratified by sex with normal versus abnormal endothelial-independent and endothelial-dependent microvascular function. [file 12933_2019_833_MOESM1_ESM.docx]

**Table 1a**

| ***Males*** | **CFRAdn Ratio**  **> 2.5**  **N=39 (75%)** | **CFRAdn Ratio**  **≤ 2.5**  **N=13 (25%)** | *P Value* | **%ΔCBFAch > 50%**  **N=16 (30.8%)** | **%ΔCBFAch ≤ 50%**  **N=36 (69.2%)** | *P Value* |
| --- | --- | --- | --- | --- | --- | --- |
| Age, years (SD) | 54.3 (10.1) | 61.6 (8.0) | 0.013 | 49.8 (10.8) | 58.9 (8.5) | 0.006 |
| BMI, kg/m^2^ (SD) | 32.6 (6.4) | 32.0 (5.8) | 0.753 | 32.0 (7.7) | 32.7 (5.5) | 0.742 |
| Hypertension, n (%) | 26 (66.7%) | 10 (76.9) | 0.480 | 10 (62.5) | 26 (72.2) | 0.487 |
| Hyperlipidemia, n (%) | 29 (74.4%) | 10 (76.9%) | 0.852 | 13 (81.3%) | 26 (72.2%) | 0.480 |
| History of MI, n (%) | 3 (7.7%) | 2 (15.4%) | 0.502 | 2 (12.5%) | 3 (8.3%) | 0.787 |
| History of Vascular Disease, n (%) | 5 (12.8%) | 0 (0.0%) | 0.172 | 1 (6.3) | 4 (11.1%) | 0.386 |
| Smoking Status, n (%)  Never Smoked  Former Smoker  Current Smoker | 11 (28.2%)  23 (59.0%)  4  (10.3%) | 5  (38.5%)  6  (46.2%)  2  (15.4%) | 0.705 | 4  (25.0%)  10  (62.5%)  2  (12.5%) | 12  (33.3%)  19  (52.8%)  4  (11.1%) | 0.754 |
| Total Cholesterol, mg/dL (SD) | 173.6 (52.1) | 149.8 (31.5) | 0.060 | 177.9 (60.5) | 162.9 (42.5) | 0.394 |
| HDL-C, mg/dL (SD) | 44.9 (15.2) | 44.2 (13.1) | 0.873 | 41.7 (9.8) | 46.0 (16.1) | 0.255 |
| LDL-C, mg/dL (SD) | 97.1 (39.4) | 80.5 (29.3) | 0.123 | 102.9 (43.8) | 88.1 (34.0) | 0.258 |
| Triglycerides, mg/dL (SD) | 156.9 (110.2) | 126.4 (54.3) | 0.202 | 164.0 (130.4) | 142.5 (83.7) | 0.564 |
| Insulin,  mg/dL (SD) | 20.0 (34.5) | 78.5 (193.3) | 0.365 | 14.7 (12.4) | 41.0 (114.6) | 0.216 |
| BNP, pg/dL (SD) | 33.4 (48.8) | 69.8 (89.4) | 0.278 | 22.2 (13.3) | 51.9 (72.9) | 0.082 |
| Heart rate, bpm (SD) | 70.5 (13.9) | 63.8 (8.2) | 0.046 | 71.1 (16.4) | 67.8 (11.4) | 0.485 |
| Systolic Blood Pressure, mmHg (SD) | 138.2 (21.4) | 137.8 (18.7) | 0.948 | 137.1 (16.5) | 138.5 (22.2) | 0.814 |
| Diastolic Blood Pressure, mmHg (SD) | 77.3 (11.3) | 72.2 (18.8) | 0.362 | 77.5 (10.0) | 75.4 (14.9) | 0.572 |
| CFRAdn Ratio, (SD) | 3.2 (0.5) | 2.2 (0.3) | <0.001 | 3.0 (0.4) | 2.9 (0.7) | 0.475 |
| %ΔCBFAch, (SD) | 34.6 (75.3) | 17.3 (97.3) | 0.567 | 122.5 (79.1) | -10.7 (34.4) | <0.001 |
| ***Females*** | **CFRAdn Ratio**  **> 2.5**  **N=41 (53.2%)** | **CFRAdn Ratio**  **≤ 2.5**  **N=36**  **(46.8%)** | *P Value* | **%ΔCBFAch > 50%**  **N=35 (45.5%)** | **%ΔCBFAch ≤ 50%**  **N=42 (54.5%)** | *P Value* |
| Age, years (SD) | 51.3 (10.0) | 53.6 (10.5) | 0.316 | 50.7 (11.1) | 53.8 (9.4) | 0.188 |
| BMI, kg/m^2^ (SD) | 34.2 (8.7) | 34.6 (8.0) | 0.834 | 33.5 (8.8) | 35.1 (7.9) | 0.408 |
| Hypertension, n (%) | 30 (73.2%) | 20 (55.6) | 0.106 | 22 (62.9) | 28 (66.7) | 0.727 |
| Hyperlipidemia, n (%) | 31 (75.6%) | 27 (75.0%) | 0.951 | 23 (65.7%) | 35 (83.3%) | 0.074 |
| History of MI, n (%) | 5 (12.2%) | 6 (16.7%) | 0.700 | 5 (14.3%) | 6 (14.3%) | 0.819 |
| History of Vascular Disease, n (%) | 5 (12.2%) | 4 (11.1) | 0.986 | 6 (17.1) | 3 (7.1%) | 0.386 |
| Smoking Status, n (%)  Never Smoked  Former Smoker  Current Smoker | 20 (48.8%)  16 (39.0%)  5  (12.2%) | 20  (55.6%)  14  (38.9%)  2  (5.6%) | 0.566 | 13  (37.1%)  19  (54.3%)  3  (8.6%) | 27  (64.3%)  11  (26.2%)  4  (9.5%) | 0.036 |
| Total Cholesterol, mg/dL (SD) | 185.0 (41.2) | 190.4 (45.9) | 0.606 | 185.6 (44.1) | 188.8 (42.8) | 0.756 |
| HDL-C, mg/dL (SD) | 52.0 (16.6) | 48.8 (11.4) | 0.349 | 52.8 (16.5) | 48.7 (12.6) | 0.251 |
| LDL-C, mg/dL (SD) | 98.8 (35.6) | 102.4 (40.2) | 0.704 | 99.4 (37.2) | 101.3 (38.2) | 0.836 |
| Triglycerides, mg/dL (SD) | 162.0 (98.6) | 189.3 (129.7) | 0.329 | 154.9 (108.6) | 190.5 (116.3) | 0.184 |
| Insulin,  mg/dL (SD) | 11.7 (9.0) | 20.3 (29.9) | 0.164 | 16.8 (29.7) | 15.4 (14.4) | 0.833 |
| BNP, pg/dL (SD) | 83.9 (121.1) | 65.2 (100.9) | 0.622 | 69.5 (104.2) | 79.8 (118.9) | 0.789 |
| Heart rate, bpm (SD) | 70.6 (10.6) | 78.6 (13.2) | 0.005 | 73.2 (13.1) | 75.2 (12.0) | 0.489 |
| Systolic Blood Pressure, mmHg (SD) | 134.8 (16.9) | 137.4 (22.1) | 0.576 | 134.4 (23.2) | 137.3 (15.9) | 0.546 |
| Diastolic Blood Pressure, mmHg (SD) | 71.7 (13.7) | 74.3 (14.3) | 0.417 | 71.9 (15.5) | 73.8 (12.7) | 0.584 |
| CFRAdn Ratio, (SD) | 3.2 (0.6) | 2.2 (0.3) | <0.001 | 2.8 (0.6) | 2.7 (0.8) | 0.698 |
| %ΔCBFAch, (SD) | 83.9 (110.9) | 21.9 (67.7) | 0.004 | 134.5 (87.7) | -11.4 (37.4) | <0.001 |

Summary of Clinical Characteristics Between Patients Stratified by Sex with Normal Versus Abnormal Endothelial-Independent and Endothelial-Dependent Microvascular Function

Abbreviations – BMI: Body Mass Index; BNP: Brain Natriuretic Peptide; CFRAdn Ratio: Coronary Flow Reserve Ratio in Response to Adenosine; HDL-C: High Density Lipoprotein Cholesterol; LDL-C: Low Density Lipoprotein Cholesterol; MI: Myocardial Infarction; %ΔCBFAch: Percentage Change In Coronary Blood Flow in Response to Acetylcholine

**Table 2b**

|  | **CFRAdn Ratio**  **> 2.5** | **CFRAdn Ratio**  **≤ 2.5** | *P Value* | **%ΔCBFAch > 50%** | **%ΔCBFAch ≤ 50%** | *P Value* |
| --- | --- | --- | --- | --- | --- | --- |
| ***Males*** |  |  |  |  |  |  |
| Metformin, n (%) | 14 (35.9%) | 3 (23.1%) | 0.383 | 5 (31.3%) | 12 (33.3%) | 0.882 |
| Thiazolidinedione, n (%) | 4 (10.3%) | 3 (23.1%) | 0.264 | 1 (6.3%) | 6 (16.7%) | 0.280 |
| Sulfonylurea, n (%) | 7 (18.0%) | 2 (15.4%) | 0.831 | 4 (25.0%) | 5 (13.9%) | 0.340 |
| Meglitinides, n (%) | 0 (0.0%) | 0 (0.0%) | - | 0 (0.0%) | 0 (0.0%) | - |
| Dipeptidyl Peptidase-4 Inhibitors, n (%) | 1 (2.6%) | 0 (0.0%) | 0.446 | 0 (0.0%) | 1 (2.8%) | 0.388 |
| Glucagon-like Peptide 1 Analog, n (%) | 2 (5.1%) | 0 (0%) | 0.069 | 2 (12.5%) | 0 (0.0%) | 0.027 |
| Insulin, n (%) | 7 (18.0%) | 7 (53.9%) | 0.015 | 3 (18.8%) | 11 (30.6%) | 0.365 |
| Dihydropyridines, n (%) | 10 (25.6%) | 3 (23.1%) | 0.852 | 5 (31.3%) | 8 (22.2%) | 0.493 |
| Diltiazem, n (%) | 8 (20.5%) | 0 (0.0%) | 0.024 | 3 (18.8%) | 5 (13.9%) | 0.659 |
| Statins, n (%) | 24 (61.5%) | 8 (61.5%) | 1.000 | 10 (62.5%) | 22 (61.1%) | 0.924 |
| Ranolazine, n (%) | 1 (2.6%) | 0 (0.0%) | 0.446 | 1 (6.3%) | 0 (0.0%) | 0.121 |
| ACE-Inhibitors or  ARBs, n (%) | 21 (53.9%) | 9 (69.2%) | 0.325 | 11 (68.8%) | 19 (52.8%) | 0.028 |
| Beta Blockers, n (%) | 12 (30.8%) | 7 (53.9%) | 0.140 | 8 (50.0%) | 11 (30.6%) | 0.183 |
| Aspirin, n (%) | 27 (69.2%) | 8 (61.5%) | 0.612 | 10 (62.5%) | 25 (69.4%) | 0.624 |
| L-Arginine, n (%) | 2 (5.1%) | 0 (0.0%) | 0.278 | 2 (12.5%) | 0 (0.0%) | 0.027 |
| Diuretics, n (%) | 9 (23.1%) | 4 (30.8%) | 0.285 | 4 (25.0%) | 9 (25.0%) | 1.000 |
| Nitrates, n (%) | 12 (30.8%) | 3 (23.1%) | 0.590 | 4 (25.0%) | 11 (30.6%) | 0.681 |
| ***Females*** |  |  |  |  |  |  |
| Metformin, n (%) | 14 (34.2%) | 15 (41.7%) | 0.497 | 11 (31.4%) | 18 (42.9%) | 0.301 |
| Thiazolidinedione, n (%) | 1 (2.4%) | 1 (2.8%) | 0.926 | 1 (2.9%) | 1 (2.4%) | 0.896 |
| Sulfonylurea, n (%) | 5 (12.2%) | 7 (19.4%) | 0.382 | 3 (8.6%) | 9 (21.4%) | 0.113 |
| Meglitinides, n (%) | 0 (0.0%) | 1 (2.8%) | 0.215 | 0 (0.0%) | 1 (2.4%) | 0.269 |
| Dipeptidyl Peptidase-4 Inhibitors, n (%) | 1 (2.4%) | 0 (0%) | 0.259 | 1 (2.9%) | 0 (0.0%) | 0.207 |
| Glucagon-like Peptide 1 Analog, n (%) | 1 (2.4%) | 0 (0%) | 0.259 | 0 (0.0%) | 1 (2.4%) | 0.269 |
| Insulin, n (%) | 9 (22.0%) | 6 (16.7%) | 0.558 | 8 (22.9%) | 7 (16.7%) | 0.500 |
| Dihydropyridines, n (%) | 5 (12.2%) | 5 (13.9%) | 0.826 | 4 (11.4%) | 6 (14.3%) | 0.709 |
| Diltiazem, n (%) | 10 (24.4%) | 7 (19.4%) | 0.601 | 11 (31.4%) | 6 (14.3%) | 0.071 |
| Statins, n (%) | 18 (43.9%) | 17 (47.2%) | 0.770 | 14 (40.0%) | 21 (50.0%) | 0.380 |
| Ranolazine, n (%) | 1 (2.4%) | 1 (2.8%) | 0.926 | 1 (2.9%) | 1 (2.4%) | 0.900 |
| ACE-Inhibitors or  ARBs, n (%) | 15 (36.6%) | 16 (44.4%) | 0.483 | 10 (28.6%) | 21 (50.0%) | 0.054 |
| Beta Blockers, n (%) | 19 (46.3%) | 14 (38.9%) | 0.509 | 12 (34.3%) | 21 (50.0%) | 0.164 |
| Aspirin, n (%) | 24 (58.5%) | 26 (72.2%) | 0.207 | 24 (68.6%) | 26 (61.9%) | 0.541 |
| L-Arginine, n (%) | 2 (4.9%) | 2 (5.6%) | 0.894 | 2 (5.7%) | 2 (4.8%) | 0.852 |
| Diuretics, n (%) | 13 (31.7%) | 17 (47.2%) | 0.163 | 10 (28.6%) | 20 (47.6%) | 0.086 |
| Nitrates, n (%) | 10 (24.4%) | 13 (36.1%) | 0.262 | 13 (37.1%) | 10 (23.8%) | 0.203 |

Summary of Medication Use at the Time of Coronary Catheterization Between Patients Stratified by Sex with Normal Versus Abnormal Endothelial-Independent and Endothelial-Dependent Microvascular Function

Abbreviations – ACE-inhibitors: Angiotensin Converting Enzyme-inhibitors; ARB: Angiotensin Receptor Blockers; CFRAdn Ratio: Coronary Flow Reserve Ratio in Response to Adenosine; %ΔCBFAch: Percentage Change In Coronary Blood Flow in Response to Acetylcholine
